# Thinking Outside the Bug: Targeting Outer Membrane Proteins for *Burkholderia* Vaccines

**DOI:** 10.3390/cells10030495

**Published:** 2021-02-25

**Authors:** Megan E. Grund, Jeon Choi Soo, Christopher K. Cote, Rita Berisio, Slawomir Lukomski

**Affiliations:** 1 Department of Microbiology, Immunology and Cell Biology, School of Medicine, West Virginia University, Morgantown, WV 26506, USA; meg0053@mix.wvu.edu (M.E.G.); sochoi@hsc.wvu.edu (S.J.C.); 2 Bacteriology Division, The United States Army Medical Research Institute of Infectious Diseases (USAMRIID), Frederick, MD 21702, USA; christopher.k.cote.civ@mail.mil; 3 Institute of Biostructures and Bioimaging, National Research Council (CNR-IBB), 80145 Naples, Italy; rita.berisio@cnr.it

**Keywords:** vaccine, *Burkholderia*, cystic fibrosis, melioidosis, glanders, outer membrane proteins, OmpA, OmpW, Omp85, Bucl8

## Abstract

Increasing antimicrobial resistance due to misuse and overuse of antimicrobials, as well as a lack of new and innovative antibiotics in development has become an alarming global threat. Preventative therapeutics, like vaccines, are combative measures that aim to stop infections at the source, thereby decreasing the overall use of antibiotics. Infections due to Gram-negative pathogens pose a significant treatment challenge because of substantial multidrug resistance that is acquired and spread throughout the bacterial population. *Burkholderia* spp. are Gram-negative intrinsically resistant bacteria that are responsible for environmental and nosocomial infections. The *Burkholderia cepacia* complex are respiratory pathogens that primarily infect immunocompromised and cystic fibrosis patients, and are acquired through contaminated products and equipment, or via patient-to-patient transmission. The *Burkholderia pseudomallei* complex causes percutaneous wound, cardiovascular, and respiratory infections. Transmission occurs through direct exposure to contaminated water, water-vapors, or soil, leading to the human disease melioidosis, or the equine disease glanders. Currently there is no licensed vaccine against any *Burkholderia* pathogen. This review will discuss *Burkholderia* vaccine candidates derived from outer membrane proteins, OmpA, OmpW, Omp85, and Bucl8, encompassing their structures, conservation, and vaccine formulation.

## 1. Introduction

Following the “Golden Age” of antibiotic discovery, antibiotic resistance quickly arose in tandem, resulting in the emergence of antimicrobial resistant (AMR) bacteria. The “ESKAPE” pathogens, made up of *Enterococcus faecium*, *Staphylococcus aureus*, *Klebsiella pneumoniae*, *Acinetobacter baumannii*, *Pseudomonas aeruginosa*, and *Enterobacter* spp., were recognized by the Centers for Disease Control and Prevention as the highly multidrug resistant bacteria of the greatest concern [1]. In addition, multidrug resistant *Burkholderia* spp., responsible for infections in patients with cystic fibrosis or melioidosis, are of rising concern due to their intrinsic multidrug resistance, increased globalization, and problems with identification, reporting, and treatment [2,3,4]. Therefore, prophylactic medical countermeasures such as vaccines, are an attractive treatment option against MDR bacteria to stop infection before it starts. In this review, we will highlight outer membrane proteins (OMPs) as vaccine targets of *Burkholderia*-derived antigens.

## 2. *Burkholderia* Infections

The *Burkholderia* spp. form a genus of Gram-negative, predominantly soil dwelling bacteria made of three monophyletic clades that consist of human, animal, and plant pathogens [5]. Two main clades of *Burkholderia* spp. are of direct health concern to humans, e.g., the *Burkholderia pseudomallei* complex (Bpc) and the *Burkholderia cepacia* complex (BCC), while a third clade incorporates phytogenic species. These intracellular bacteria can cause respiratory infections, where the bacteria invade airway epithelial cells or pulmonary macrophages with varying effectiveness [6]. As a part of their intracellular lifestyle, *Burkholderia* spread from cell to cell by fusing neighboring cells and forming cell conglomerates known as multinucleated giant cells (MNGC). MNGCs can be used as a hallmark of infection, and postulated to be involved in formation of granuloma-like lesions [7,8,9,10]. Antibiotic resistance in *Burkholderia* is determined by multiple mechanisms, for example the production of class A PenA β-lactamases leads to ceftazidime and amoxicillin–clavulanic acid resistance. Another mechanism is attributed to the abundance of porins and efflux pumps in *Burkholderia*, specifically those of the resistance nodulation division family [4]. These proteins and protein complexes are known to add stability to the cell membrane and increase resistance to aminoglycosides, tetracycline, macrolides, chloramphenicol, fluoroquinolones, trimethoprim and trimethoprim–sulfamethoxazole [11]. Pathogenic *Burkholderia* have a modified lipid A portion of their lipopolysaccharide (LPS) that decreases the net negative charge of the bacterial cell, which ultimately decreases susceptibility to cationic antimicrobial peptides and polymyxins [4,12]. Due to these factors, *Burkholderia* infections are still a formidable threat.

### 2.1. Burkholderia Pseudomallei Complex

The Bpc consists of *Burkholderia pseudomallei*, *Burkholderia mallei*, and non-pathogenic *Burkholderia thailandensis*. *B. pseudomallei* and *B. mallei* are classified in the United States as Tier one select agents, because they have a potential to be used as bioweapons, due to the pathogens’ intrinsic multidrug resistance, ability to be aerosolized, and lack of a vaccine [13]. Clinically, *B*. *pseudomallei* is the causative agent of melioidosis, a neglected tropical disease that is endemic in countries such as Thailand, Vietnam, India, and Australia, but is underreported in many other equatorial regions [14]. Compared globally to other neglected tropical diseases, melioidosis has a high disability-adjusted life years (DALYs), which is a measure of disease burden that takes in account the life years lost due to infection [15]. Transmission mainly occurs via aerosolization of the bacteria (e.g., aerosolized by driving rains during monsoon seasons or other weather events) or through direct contact with contaminated soil and water sources [16,17,18]. *B. pseudomallei* affects the pulmonary and cardiovascular systems, with the main symptoms being pneumonia and sepsis, but can also result in epithelial lesions and ulcers, or neurological defects [3]. Similarity of symptoms to other diseases has led to *B. pseudomallei* being termed the “great mimicker”, which is often misdiagnosed as other infections such as tuberculosis [19]. Untreated melioidosis has a mortality rate approaching 90%, which decreases to 50% with treatment. Additionally, *B. pseudomallei* can lead to latent/chronic infections, with some infections reemerging >20 years later [20,21], which reduces identification and treatment [3]. *B*. *mallei*, a closely-related clonal derivative of *B. pseudomallei*, causes the disease glanders in horses and related livestock, and can be fatal if contracted by humans [22]. Although *B. thailandensis* is considered non-pathogenic, it has been documented as the causative agent in a few patient cases that involved traumatic injury [23,24,25]. Because it is not a health concern or biothreat, *B. thailandensis* has been used as Biosafety Level 2 surrogate organism for Biosafety Level 3 select agents *B. pseudomallei* and *B. mallei* [26,27,28,29].

### 2.2. Burkholderia Cepacia Complex

The BCC is a larger clade made of more than 22 species that can cause pulmonary opportunistic infections in cystic fibrosis patients, or immunocompromised patients with genetic disorders like chronic granulomatous disease. BCC pathogens account for 5% of cystic fibrosis patient infections, and while not the source of most infections, these pathogens are still a public health concern as the majority of BCC infections are not eradicated [30]. Of the 22 species, *B. cenocepacia* and *B. multivorans* are the most predominant pathogens globally, hovering around 70–80% of BCC infections for the past 20 years [31,32]. However, the local distribution of species can differ. For example, in the United States a phytogenic species *B. gladioli* accounts for 15% of BCC infections, which is not common in other countries [33]. In Argentina, the most common isolate from industrial contaminated products was *B. contaminans* at 42%, and it is also the etiologic agent of the most common BCC infection in that country [34]. Other species *B. vietnamiensis* and *B. dolosa* also infect more frequently, from 9–20% depending on region, while *B. anthina* and *B. stagnalis* make up less than 1% [32,33]. Transmission occurs via contact with contaminated medical equipment, water-based pharmaceuticals or hygiene products, exposure to environment, or can occur from person to person transmission [35,36]. As with melioidosis, patients can be asymptomatic or deteriorate rapidly, leading to septicemia and necrotizing pneumonia, known as *cepacia* syndrome. One of the concerning traits of the BCC is their capability to adapt to the host or compete with *P. aeruginosa*, the leading infection of cystic fibrosis patients [37].

## 3. Targeting Outer Membrane Proteins for Vaccines

The outer membrane of Gram-negative bacteria harbors important cell structures, such as porins, outer membrane proteins (OMPs), efflux pumps, and LPS that are essential for cell physiology, and resistance to antibiotics [38]. These outer membrane components are common targets for antibody-based therapeutics or vaccines for several reasons. OMPs have surface-exposed epitopes, thus, are accessible for antibody or T cell receptor recognition, and are involved in essential tasks such as adhesion, biofilm formation, regulation of quorum sensing, or extrusion of toxic substances. Additionally, these proteins can be conserved and highly expressed [39], thereby, increasing antigen availability. For example, one of the current licensed and widely distributed subunit vaccines that targets surface-exposed antigens is the acellular pertussis vaccine, which uses a combination of fimbriae, pertactin, and filamentous hemagglutinin with pertussis toxoid [40]. Diverse approaches have been developed for identifying immunogenic targets, including classical, reverse, and structural vaccinology. Classical vaccinology identifies targets on pathogens expressed in vivo or in vitro. Alternatively, reverse vaccinology uses a genomic approach for identifying surface-exposed proteins or immune cell-reactive epitopes [41], and structural vaccinology identifies possible epitopes based on structural features of antigens [42]. In the end, the goal is to select antigens that elicit a robust immune and memory responses, thus leading to a specific and long-lasting protection [43].

### 3.1. Burkholderia-Derived Vaccine Candidates

The pursuit for a licensed *Burkholderia* vaccine has been ongoing for many years, with a rise in vaccine research during the 2000s [44]. There have been many candidates that have shown potential, ranging from live attenuated vaccines to subunit vaccines, however few have approached a level of effectiveness to merit consideration for clinical trials [45]. Some of the present candidates being investigated for *B. pseudomallei* and/or *B. mallei* include Hcp1, a component of a type VI secretion system, and capsular polysaccharide (CPS) [46]. It has also been demonstrated that naturally derived outer membrane vesicle vaccines offer protection against *B. pseudomallei* infection, with noted cross-protection observed against *B. mallei* infection [47,48]. Live attenuated vaccines continue to be investigated and may play a part in future vaccine strategies being evaluated [49,50,51]. More recently, a functionalized gold-nanoparticle-multivalent vaccine showed that innovative antigen formulations can enhance protection from already promising candidates [52,53], increasing some survival rates to 100% when challenged with a 50 × LD_50_ dose [53]. These experiments characterizing vaccine strategies have generated results differing in efficacy, type of immune response, and logistical feasibility. The outer membrane contains a myriad of proteins that have been identified as immunogenic and are potential vaccine targets. In this review, we discuss the OMP vaccine targets OmpA, OmpW, Omp85, and Bucl8, which all form outer membrane β-barrel structures with surface-exposed epitopes (Table 1).

### 3.2. Outer Membrane Protein OmpA

Development of an effective subunit vaccine requires identifying proteins that are abundant, well-conserved across species—or even genera—and elicit a robust, long-lasting immune response. Outer membrane protein A (OmpA) is an evolutionary conserved family of proteins found across Gram-negative bacteria, which has been well-studied and characterized in model species, such as *Escherichia coli* and *A. baumannii* [54]. The monomeric protein consists of eight antiparallel β-strands, four surface-exposed loops, and three periplasmic turns that collectively form an outer membrane β-barrel [55,56]. Functionally, OmpA plays a role in adhesion and biofilm formation [57,58], and acts as a receptor of colicin, a protein toxin produced to target members of closely related species [59]. OmpA also adds stability to the membrane through a network of salt bridges and hydrogen bonds formed by a network of charged residues that increases the thermal stability of the OmpA protein [60]. In addition, alterations of surface-exposed loops by shortening or extension, or to internal inward-facing residues and β-strand side chains did not impact β-barrel formation [55]. Therefore, the stable structure, high-level of conservation, high copy number—estimated at about 100,000 per cell– and confirmed immunogenic epitopes, makes OmpA a good vaccine target. To this point, OmpA has been evaluated as a vaccine candidate in several concerning Gram-negative pathogens, including *E. coli* [61], *A. baumannii* [62], *P. aeruginosa* [63,64], and *Burkholderia* spp. [65,66].

Bioinformatic analyses of sequenced genomes from diverse Burkholderia species have revealed the presence of numerous OmpA homologs. Of the 12 putative OmpA proteins identified in *B. pseudomallei* and tested, Omp3 and Omp7 showed immunoreactivity with pooled sera from melioidosis patients at different disease stages. Furthermore, immunization with either recombinant Omp3 or Omp7, combined with Freund’s complete adjuvant, protected 50% of mice from a lethal dose of *B. pseudomallei* [65], which is comparable to protection reported in mice immunized with LPS [67]. Immunization with Omp7 generated a greater IgG response than Omp3, but both were greatly increased compared to sera from non-immunized controls. Antibody characterization determined that the major IgG subclass of immune sera was Ig2a, with an IgG2a/IgG1 ratio of 1.2 for Omp3 and 1.3 for Omp7. These ratios indicate a Th1-driven response, which are targeted towards fighting intracellular infections [65]. However, Freund’s adjuvant is not approved for human use due its toxicity/reactogenicity, and therefore an alternative would need to be tested. While vaccination with these proteins did not provide full protection in this pilot study, combining Omp3 and Omp7 with other adjuvant or antigens could help boost the immune response to provide further protection in future studies.

A similar approach was also used in a study aimed at identifying vaccine candidates by employing immune sera from cystic fibrosis patients with ongoing BCC infections. Infected cystic fibrosis patients seroconverted to BCAL2958, an OmpA-like protein identified in *B. cenocepacia* J2315 and conserved within other BCC isolates [68]. A recombinant protein of BCAL2958 was immunoreactive with four sera samples from patients with confirmed *B. cenocepacia* infections, but not with sera from healthy donors. Protein BCAL2958 and homologs present in *B. cenocepacia* isolates and related BCC species shared at least 96% sequence similarity, indicating BCAL2958 is a conserved protein with potential for cross-species protection in vaccinated population. Further demonstrating cross-reactivity, a western blot analysis with anti-recombinant BCAL2958 antibodies recognized immunoreactive bands in cell lysates prepared from six different BCC species. A recent study demonstrated in vitro neutrophil activation by OmpA in the presence of Th17 cytokines, which are involved in mucosal defenses and activation of neutrophils during inflammation. The result was increased levels of TNF-α, H_2_O_2_, and catalase, indicating a possible role for neutrophils and Th17 immunity during *Burkholderia* infection [69]. Further work from members of this group has employed a technique termed “surface shaving”, which uses partial proteolysis of the surface-exposed proteins, and in theory proteins that are exposed and accessible to the immune system, which then can be analyzed by mass spectrometry [70]. Identification of immunogenic antigens is a valuable steppingstone towards vaccine development; however, it does not predict whether the antigen is protective, and thus follow-up investigations are necessitated.

### 3.3. Outer Membrane Protein OmpW

Like OmpA, OmpW is a common OMP found in Gram-negative bacteria, and evidence of its immunogenicity dates back to 1980′s, when it was first described as immunoreactive determinant with sera from patients infected with *Vibrio cholerae* [71,72]. Later the *E. coli* protein was crystallized and the structure was solved at 2.7 Å resolution, although, the function was not immediately determined [73]. Structurally, OmpW forms an eight-stranded β-barrel situated within the outer membrane, with loops extended into the extracellular space. Within the β-barrel of the crystallized OmpW is a hydrophobic gate comprised of two residues Leu and Trp, modulating the transport of hydrophobic compounds. It was hypothesized that OmpW acts as a transport protein or porin for hydrophobic molecules, as well as a colicin S4 receptor [73].

The structure of OmpW in *Burkholderia* spp. has not been solved as of yet, however the protein has been purified and tested in immunized mice for protection against *B. multivorans* and *B. cenocepacia* infections [74]. Two adhesins, linocin and OmpW, were identified as immunoreactive proteins with sera from cystic fibrosis patients with infections by these pathogens [74]. Immunization with OmpW significantly reduced bacterial burden by several logs in the lungs of mice infected with BCC organisms. Antigens that are upregulated during infection or necessary for virulence or survival are often conserved. Protection was also transferred to *B. pseudomallei* when mice were immunized with the recombinant protein BpOmpW and tested in two different challenge models, representing acute and chronic melioidosis [75]. Immunization significantly extended survival from infection in both models, and protected 75% of mice for over 80 days [75]. Both BCC and Bp models demonstrate that OmpW is a potential protective antigen, and modulation of the vaccine formulation could increase protection to sterilizing immunity.

Protection against a pathogen can sometimes be elicited by a single antigen, but if the antigen is not highly immunogenic, the immune response can be enhanced with protein conjugation. One of the emerging technologies in the vaccine field is conjugating proteins to nanoparticles to enhance immunogenicity by stimulating specific immune responses, decreasing degradation, and increasing binding or uptake of the bacterium [76]. A recent study utilized gold-nanoparticles (AuNP) and LPS conjugated to previously recognized immunogenic antigens (represented as AuNP-protein-LPS), including OmpW, Hcp1, OpcP, OpcP1, FlgL or hemagglutinin, in a murine glanders model [53]. Of the six single-component conjugations tested, only OmpW and OpcP1, a subunit of the outer membrane porin OpcPO, had a 100% survival rate when mice were intranasally challenged with two LD_50_ doses of *B. mallei*. Interestingly, a mix of the six antigens provided less protection, at 80% survival, than that of OmpW alone, which could be due to the diluted concentration of each antigen compared to the single or trivalent formulations. However, there were still bacteria present in all the spleens of mice immunized with AuNP-OmpW-LPS, and in the lungs of ~50% of the mice. When challenged with a greater dose, 50 times the LD_50_, immunization with AuNP-OmpW-LPS provided protection from death for 80% of the mice, while the combination of OmpW, hemagglutinin, and OpcP increased protection to 100%. This study demonstrates substantial progress in developing a vaccine for glanders, and potentially protection will be afforded to animals infected with *B. pseudomallei* in future studies.

### 3.4. Outer Membrane Protein Omp85

Similar to OmpA and OmpW, Omp85-family proteins are outer membrane β-barrels that are highly conserved amongst Gram-negative bacteria. In contrast to the aforementioned antigens, the general structure of Omp85 consists of two main domains: (i) a C-terminal β-barrel made of 12–16 antiparallel β-strands, and (ii) one to five polypeptide-transport-associated (POTRA) domains [77]. Functionally, Omp85 is a constituent of a two-component system that inserts proteins or LPS into the outer membrane. Supplementary components include inner membrane secretion proteins, such as SecYEG, that translocate the unfolded protein from the cytoplasm to the periplasm, and chaperones like Skp and SurA that shuttle the protein/lipids to Omp85 for assembly. The Omp85-family includes essential proteins BamA, of which anti-BamA monoclonal antibody have been shown to inhibit the growth of *E. coli* [78], or FhaC, the transporter for filamentous hemagglutinin of *Bordetella* [79].

The Omp85-family antigen BPSL2151, was identified as an immunogenic protein expressed in patients with *B. pseudomallei* infections [80,81]. The protein was demonstrated to be conserved amongst *Burkholderia* spp., with >86% identity. Sera analysis from patients with melioidosis demonstrated antibody recognition of recombinant Omp85, while control serum from non-infected patient did not. Immunization of mice with rOmp85, significantly increased the percent survival from 10% recorded for non-immunized controls to 70% in immunized cohorts. Functional analysis of the antibodies from immune sera demonstrated increased bactericidal activity in the presence of complement or polymorphonuclear leukocytes, indicating the antibodies increased killing by classical complement pathway or opsonization [81].

**Table 1:** Vaccination candidates.

Protein Family	Protein	Species	Strain	Source
OmpA	Omp3 and Omp7	*B. pseudomallei*	K96243	[65]
	BCAL2958	*B. cenocepacia*	J2315	[68]
OmpW	OmpW	*B. cenocepacia* *B. multivorans*	BC7 LMG13010	[74]
	BpOmpW	*B. pseudomallei*	K96243	[75]
Omp85	BPSL2151/rOmp85	*B. pseudomallei*	D286	[81]
OEP	Bucl8	*B. pseudomallei*	K96243 1026b	[82]
		*B. mallei*	CLH001	

### 3.5. Outer Membrane Efflux (OEP) Protein Bucl8

**Figure 1:**
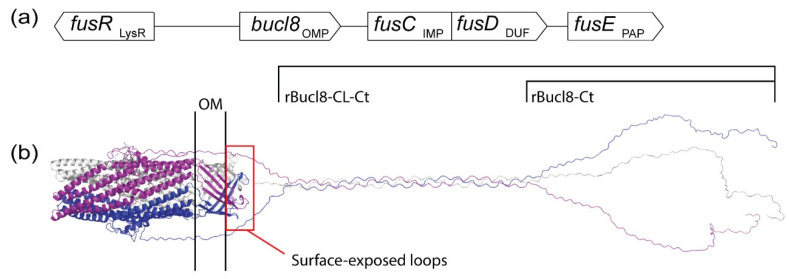
Bucl8 modeling and immunogenicity of Bucl8-derived antigens. (**a**) Schematic of genes in *bucl8* operon. Indicated gene products: LysR, LysR-type transcriptional regulator; OMP, outer membrane protein; IMP, inner membrane protein; DUF, domain of unknown function; PAP, periplasmic adaptor protein. (**b**) Homology model of Bucl8 variant in *B. pseudomallei* and *B. mallei*. Bucl8 trimeric structure is shown, with chains colored in blue, grey, and magenta. Bacterial outer membrane (OM) and the surface-exposed loops of Bucl8 are marked. Bucl8-derived recombinant proteins rBucl8-CL-Ct, which includes both the triple helical collagen-like domain (CL) and C-terminal domain (Ct), and rBucl8-Ct, containing only the unstructured C-terminal domain, are indicated. Homology modelling was performed with MODELLER, using the VceC structure (PDB code 1yc9) as a template for the periplasmic/outer membrane component and the high-resolution structure of the collagen-like peptide (PDB code 1k6f) as a template for the CL region.

*Burkholderia* collagen-like protein 8, Bucl8, is a predicted outer membrane component of an efflux pump that was recently shown to be involved in fusaric acid (FA) and *p*-hydroxybenzoic acid (pHBA) resistance [82,83]. The gene that encodes Bucl8, *bucl8*, is located in an operon consisting of the downstream genes *fusCD*, encoding the inner membrane protein (IMP) and protein of unknown function (DUF) and *fusE*, encoding the periplasmic adaptor protein (PAP) (Figure 1a). Together these components form a putative tetrapartite resistance-nodulation-division-like efflux system. Addition of FA or pHBA increased the transcription of the genes in *bucl8* operon and of the operon’s regulator, *fusR*, which is a LysR-type transcriptional regulator. When the pump was chromosomally deleted, the mutant *B. pseudomallei* demonstrated a decrease in minimum inhibitory concentration to FA and pHBA. The spectrum of substrates of Bucl8-associated efflux pump has not been fully defined. Nevertheless, the transport of pHBA suggests other aromatic compounds as substrates, for example, *p*-aminobenzoic acid, benzoate, or salicylate that are used in foods and pain-relieving drugs. While the mycotoxin FA is not ideal for administration, the non-toxic compounds found in foods and drugs could potentially upregulate Bucl8 expression during infection, thereby increasing availability of the vaccine targets.

Two homology models of Bucl8 found in *B. pseudomallei* and *B. mallei* were generated, based on the solved structures of the outer membrane proteins OprM (PDB code 1wp1) [83] and VceC (1yc9) [82], which are components of the efflux pumps in *P. aeruginosa* and *V. cholerae*, respectively. Bucl8 is a predicted trimeric outer membrane lipoprotein beginning with an N-terminal Cys residue for the attachment of the lipid moiety. The tertiary structure forms a α-helical barrel spanning the periplasm and a β-barrel traversing the outer membrane. In addition, it harbors an extended extracellular region, as depicted in Figure 1b. The extracellular portion of Bucl8 protrudes from bacterial cell as a triple-helical collagen-like (CL) domain and the carboxyl-terminal (Ct) region. The repeating (Gly-Ala-Ser or GAS)_n_ triplets form the CL domain that differs in length in Bucl8 variants from different *B. pseudomallei* and *B. mallei* strains, with a median number of 20 GAS repeats identified among ~100 query results. Circular dichroism analyses confirmed the formation of the collagen triple helix by the recombinant rBucl8-CL-Ct construct, while the rCt polypeptide was unstructured [82]. Immunization with recombinant proteins rBucl8-CL-Ct and rBucl8-Ct, corresponding to the CL-Ct or only Ct region, respectively (Figure 1b), and in combination with an adjuvant (AddaVax; Ad), elicited a specific IgG antibody response in CD-1 mice, demonstrating their potential as immunogens (manuscript in preparation).

Each Bucl8 monomer has two distinct loops (L1 and L2) on the β-barrel that are predicted to be surface-exposed and are 100% conserved in Bucl8 variants among *B. pseudomallei* and *B. mallei* (Figure 1b). CD-1 mice were vaccinated with synthetic peptides corresponding to loops L1 and/or L2 conjugated to the carrier protein, a genetically inactivated diphtheria toxoid, CRM_197_. Analysis of sera after three immunizations demonstrated generation of antigen-specific IgG antibodies (manuscript in preparation). Furthermore, the AddaVax adjuvant greatly improved the humoral response. Peptide L1 had a significantly greater antibody response compared to L2. The sera from mice immunized with mixed peptides L1 and L2 showed mixed response to L1- and L2-loop antigens. Surface-exposed loops are attractive vaccine targets, as previously discussed with the immunogenic epitopes of OmpA and OmpW.

### 3.6. Burkholderia Cepacia Complex- Bucl8-BCC Variant

**Figure 2:**
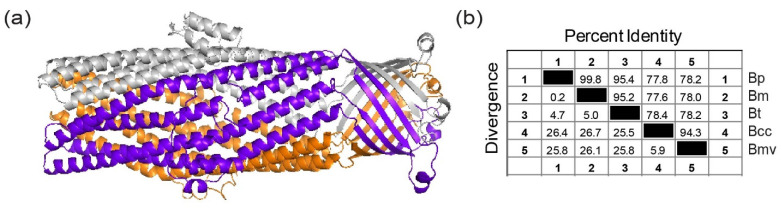
Identification and modeling of the Bucl8 variants in *Burkholderia cepacia* complex (BCC) organisms. (**a**) Bucl8-BCC variant lacks extracellular CL-Ct domain. Homology model of Bucl8-BCC protein from *Burkholderia cenocepacia* J2315 was generated with MODELLER using the structure of VceC (PDB code 1yc9, seqid 35%) as a template. (**b**) Sequence conservation of Bucl8-like proteins. Similarity matrix displays the Bucl8-sequence identity and divergence of *B. pseudomallei* (Bp) 1026b, *B. mallei* (Bm) ATCC 23344, *B. thailandensis* (Bt) E264, *B. cenocepacia* (Bcc) J2315, and *B. multivorans* (Bmv) ATCC BAA-247.

*Burkholderia cepacia* complex species harbor the Bucl8 ortholog, designated Bucl8-BCC, which lacks the extracellular CL-Ct region. The Bucl8-BCC variant in *B. cenocepacia* J2315 has a 35% protein sequence identity to the corresponding barrel regions of the VceC protein. Given the lack of structural information on this protein, we filled this gap using homology modelling and the structure of VceC as a template, Figure 2a. Similar to Bucl8 in Bpc complex species, Bucl8-BCC structure presents the characteristic periplasmic α-helical barrel and outer membrane β-strand barrel. Bucl8-BCC sequences were identified in 29 completely sequenced BCC genomes deposited in NCBI database. Multiple sequence alignment of these Bucl8-BCC variants in the region from the starting Cys residue until the end of the sequence encoding the α-helical and β-strand barrels of the mature Bucl8-Bpc protein (residues 1–459) is reported in Figure S1. Within the 41 genomes, 72.8% of residues of Bucl8-Bpc and Bucl8-BCC were conserved, and the conservation increases within each Bpc and BCC genomes. As a representation of the full alignment, Figure 2b shows the percent identity/divergence of *B. pseudomallei* (Bp) 1026b, *B. mallei* (Bm) ATCC 23344, *B. thailandensis* (Bt) E264, *B. cenocepacia* (Bcc) J2315, and *B. multivorans* (Bmv) ATCC BAA-247, which are representative BCC species with highest incidence of human infection. In the Bpc group, Bucl8 sequences of *B. pseudomallei, B. mallei,* and *B. thailandensis* have ≤5% divergence, whereas the Bucl8-BCC sequences have ≤5.9% divergence. Between all 41 isolates, there was ~78% conserved residues between Bucl8-Bpc and Bucl8-BCC sequences.

**Figure 3:**
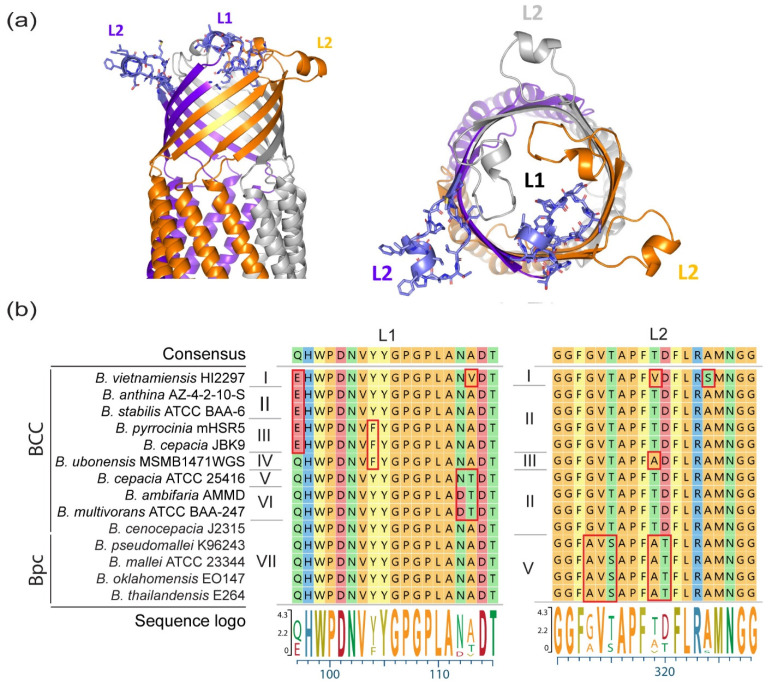
Surface exposed loops of Bucl8. (**a**) Cartoon representations (side and top views) of the outer membrane domain of *Burkholderia cenocepacia* J2315, obtained by homology modelling using MODELLER and the structure of VceC (PDB code 1yc9, seqid 35%) as a template. Surface-exposed loops L1 and L2 of each chain are highlighted. For clarity, loops L1 and L2 of chains A are drawn in blue stick representations. (**b**) Multiple sequence alignment of the loops. Variants of Bucl8-loop sequences from representative species and strains of the Bpc and BCC clades were aligned using ClustalW. Sequences were aligned from beginning of mature protein to their divergence at C-terminus, ~460 residues (Figure S1). Red boxes indicate main polymorphic regions. Roman numerals indicate loop variants.

Vaccination against conserved antigens with potential for cross-species protection would benefit a larger population of people. Figure 3a depicts the extension and potential positioning of the loops from the Bucl8-BCC β-barrel, as well as the loop 1–2 side chains. The primary sequences of the surface exposed loops are relatively conserved, with loop L1 having 15 of 19 residues identical amongst the Bpc and BCC species and loop L2 having 14 of 19 conserved residues. In the presented loop-L1 sequences there are seven variants, labeled I-VII in Figure 3b. Variants I-III have a single Q107E glutamine to glutamate substitution, compared to variants IV-VII; importantly, loop L1-residue 107 may not be fully surface-exposed, according to Bucl8-BCC model. All Bpc species have the same loop 1 sequence (variant VII), which is also shared with *B. cenocepacia* J2315, while *B. ubonensis* differs at a single position Y114F. Bucl8-BCC variant V differs from Bucl8-Bpc variant by A123T substitution, while variant VI has an additional polymorphism N122D; conserved residues 122–123 are also not at the apex of the loop 1 structure. Overall, there are two main variants of loop L2, one for Bucl8-Bpc (variant L2-V) and one for Bucl8-BCC (L2-II); two additional polymorphisms are present at position 319 and constitute variants L2-I and L2-III in Bucl8-BCC. Because BCC-infections are predominantly caused by *B. cenocepacia* and *B. multivorans* (>80% of infections [84]), that share identical L2-II sequence—and the overall high level of conservation of Bucl8, unique polymorphisms may not affect immunization efficacy with L1 and L2 against most *Burkholderia* species.

## 4. Vaccine Challenges

### 4.1. Off-Target Effects

OMP proteins that are present in pathogenic Gram-negative bacteria may share sequence similarity with their orthologs found in beneficial micro-flora. For example, OmpA is a ubiquitous, highly abundant, conserved outer membrane structure. Notably, the level of amino acid conservation within the four extracellular loops differs between genera [85,86]. The most conserved amino acid sequences of the highly conserved β-barrel structure among Omp85-like proteins are within the predicted β-strands, while the interstrand sequences of the connecting loops vary between genera. Phylogenetic analysis separates Omp85-like proteins into their respective classes of bacteria [77]. As for Bucl8, a BLASTp search on NCBI database determined the protein sequences of loops L1 and L2 were unique to *Burkholderia*.

Cross-reactivity with human proteins should also be taken under consideration. For example, Omp85-family proteins are found in Gram-negative and Gram-positive bacteria, but eukaryotic homologs are found in fungi, plants, and animals, including human mitochondria. The human mitochondrial homologs of Omp85, TOM40 and SAM, cluster with Alphaproteobacteria; however, *Burkholderia* are in a more distant clade that is a part of Betaproteobacteria [77,87]. In the case of Bucl8-Bpc, a portion of loop 1 sequence partially matched with sequences from human Ig junction region, although, the significance scores suggest the match would be inconsequential. Human collagens do not use (GAS)_n_ triplets found in the collagen-like extracellular domain of Bucl8. Furthermore, bacterial collagens differ from human collagen both in primary sequence and post-translational modifications; human collagen displays preponderance of GXY repeats containing prolines at position X and hydroxyprolines, which are not found in bacteria, at position Y [88,89,90]. The lack of cross reactivity between human collagens types I, III, and V and a collagen-like protein BclA found in spore exosporium of *Bacillus anthracis* was demonstrated by the authors by ELISA [91].

### 4.2. Epitope Accessibility and Conformation

The outer membrane is composed of a multitude of proteins, lipids, and sugars that work together to control permeability in and out of the cell. LPS covers the majority of the cell’s surface, forming gaps where the outer membrane entities are. The O-antigens in LPS are made up of a variable number of repeated oligosaccharide units—sometimes >100 in number—and this could restrict access of molecules to the bacterial surface. In *Burkholderia*, the number of O-antigens varies [92], but most likely the structure is longer than surface-exposed loops in OMPs. However, OMPs leave “footprints” in the LPS monolayer that affect antibody accessibility. A study comparing the immunogenicity of OmpA and a trimeric protein, OmpD, in *Salmonella* demonstrated that the gap left by OmpA was not large enough to fit the 50 Å width of the IgG Fab portion. Nonetheless, OmpD generated a 70 Å gap, which was correlated with increased protection when used as a vaccine antigen [93]. For example, vaccination with synthetic conjugate peptides, corresponding to surface-exposed loops of the iron receptor HpvA, have shown to elicit an immune response and provide protection against the closely related pathogen *P. aeruginosa* [94]. A molecular dynamics study of six proteins from *E. coli*, including OmpA, concluded that the O-antigens do not block access to the proteins in the OM-outer leaflet [95]. Specifically, for Bucl8, the collagen-like domain, depending on the number of (GAS)_n_ repeats, could exceed the length of LPS-O chains.

Structural vaccinology employs structural biology approach to design and re-design proteins to engineer recombinant constructs that display protective determinants, rather than the whole protein sequence. Simplified immunogenic constructs are designed to achieve high production yields, making recombinant proteins an economic and effective option for vaccine use. The recombinant antigens discussed in this review are still in their early pre-clinical stages.

The synthetic peptide vaccines can be produced rapidly, in controlled conditions, and customized to the target pathogen. However, the length of the synthetic peptide may increase the production cost significantly. In addition, linear peptides may not be as effective as whole-protein antigens if the epitope is conformation-dependent. One solution is to design cyclic peptides that resist serum proteases longer than linear peptides and adopt a more loop-like conformation, which has been demonstrated to increase immunogenicity [86,92]. Also, determining if the peptide sequence matches immunogenic epitopes is important. Bucl8-Bpc L1 and L2 have predicted MHC and TCR epitopes, and L1 in particular is predicted to be antigenic.

### 4.3. Epitope Mutation and Deletion

Conserved, essential proteins are frequent vaccine targets because mutations that compromise their function or full deletions are less likely. OmpA [54,96,97], Omp85 [77], and Bucl8 [82] affect cell survival and/or growth. OmpW increases survival in hypersaline environments [98] and decreases rate of phagocytosis [99]. Multivalent vaccines can be designed to include numerous protein variants [100]. Likewise, multi-component subunit vaccines targeting several proteins effectively combat pathogens that cannot alter all the targets [101,102].

## 5. Final Remarks

**Figure 4:**
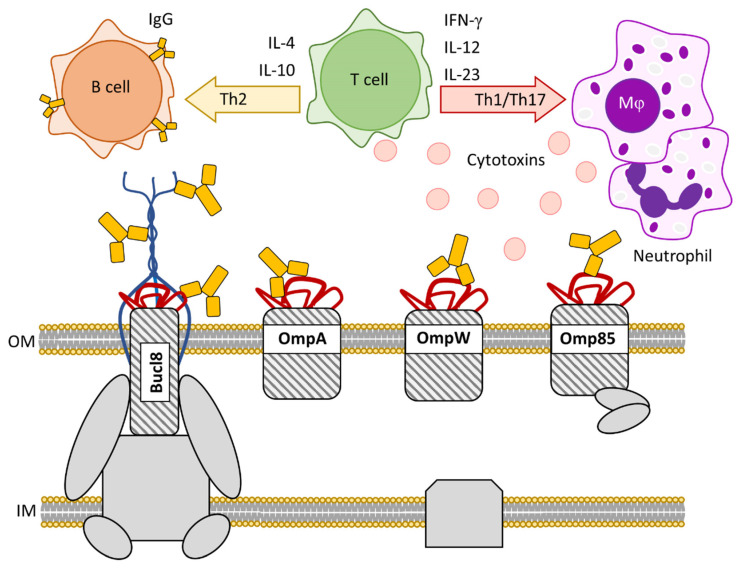
Utilizing *Burkholderia* antigens derived from outer membrane proteins for vaccine targets. OmpA, OmpW, Omp85, and Bucl8 are the outer membrane components of bacterial transport systems in *Burkholderia* spp. that have been previously identified as vaccine targets. OMPs are exploitable antigens due to surface-exposed loops and extended structures, as found in Bucl8. OMP-derived products stimulate long-lasting Th1/Th17/Th2 immune responses that augment opsonophagocytic and cytotoxic activities against invading *Burkholderia* pathogens.

(I) Novel vaccine strategies must balance a number of factors, including efficacy, safety, and costs, as well as be tailored to the target pathogen(s). In general, subunit vaccines are regarded as safer because they do not have the risk of mutants reverting to virulent phenotypes (a potential concern when using live attenuated vaccines), or toxicity associated with compounds like LPS. Acellular vaccines can also be designed to elicit a specific, homogenous immune response. OMPs are attractive targets for generating humoral responses because they are accessible for antibody binding and have been tested for robust expression (Figure 4).

(II) In this review, we have discussed vaccine targets OmpA [65,68], OmpW [73,74,75], Omp85 [81], and Bucl8 [82], which all contain outer membrane β-barrel structures that have surface-exposed epitopes and are highly conserved among the *Burkholderia* spp. Humoral responses were stimulated for all antigens, indicated by increased antigen-specific antibody titers, and provided partial–to–full protection from death in animal models.

(III) Bucl8 is a novel trimeric outer membrane efflux protein harboring the characteristic outer membrane β-barrel with two distinct surface-exposed loops in each monomer. Bucl8 variant in *B. pseudomallei* and *B. mallei* also contains an extracellular domain that extends from the bacterial cell surface [82]. Preliminary immunization data from mice immunized with synthetic-loop peptides conjugated to diphtheria toxoid, or with recombinant protein resembling the Bucl8 extracellular region, elicited robust IgG responses. Molecular modeling of the Bucl8 orthologs present in BCC organisms has identified analogous, conserved, surfaced-exposed loops as potential immunogenic targets, thus, appreciably extending the spectrum of anti-Bucl8 vaccine for pathogenic *Burkholderia* species.

(IV) Although many of the past vaccine candidates were immunogenic and showed some level of protection, their value may extend as an alternative antibody-based therapy. The use of monoclonal antibodies that are protective against target pathogens or as vehicles for targeted delivery of antimicrobials is a rapidly growing field in medicinal and countermeasure technologies. In addition, current technologies developed numerous IgG-like scaffolds that are alternative to mAbs, such as monobodies, peptibodies, monomeric Fc-fusions, or bispecific antibodies [103,104,105].

## Supplementary Materials

The following are available online at https://www.mdpi.com/2073-4409/10/3/495/s1, Figure S1: Multiple sequence alignment of α-helical and β-strand barrels of Bucl8-Bpc and Bucl8-BCC variants.
