# Supplementary figures and images for: Thinking Outside the Bug: Targeting Outer Membrane Proteins for Burkholderia Vaccines
Source: Cells. 2021 Feb 25;10(3):495. doi: 10.3390/cells10030495 (PMC7996558; doi:10.3390/cells10030495)

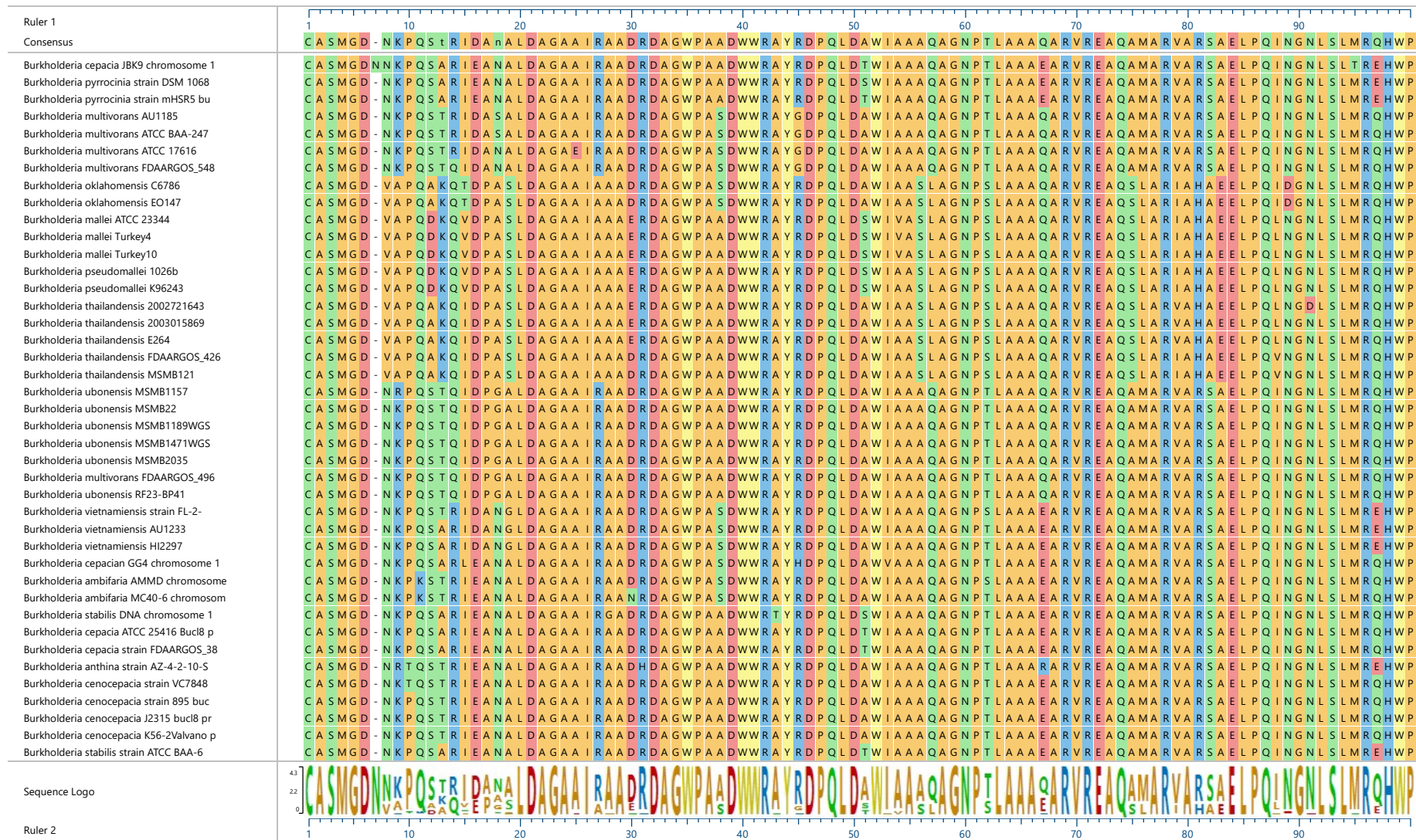

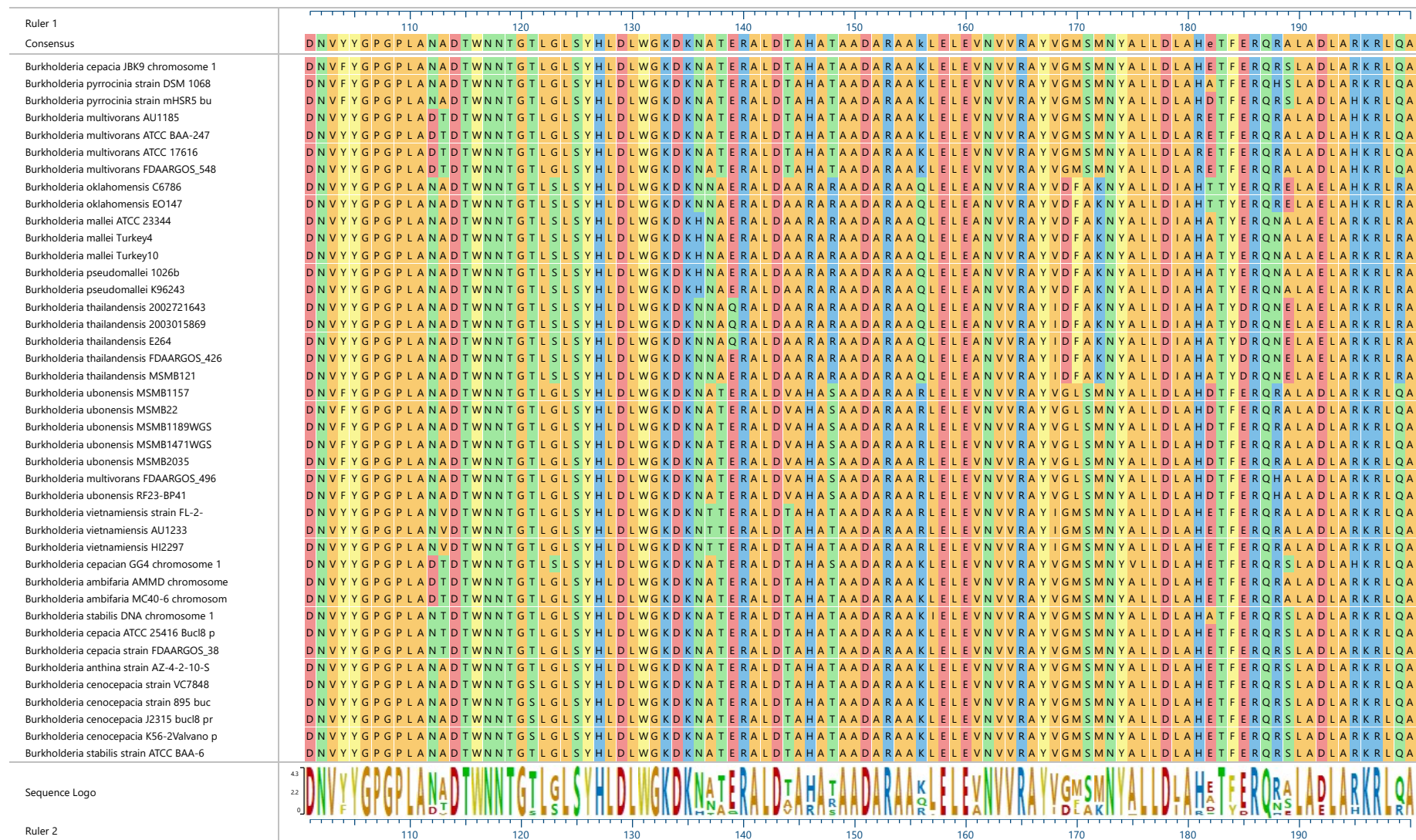



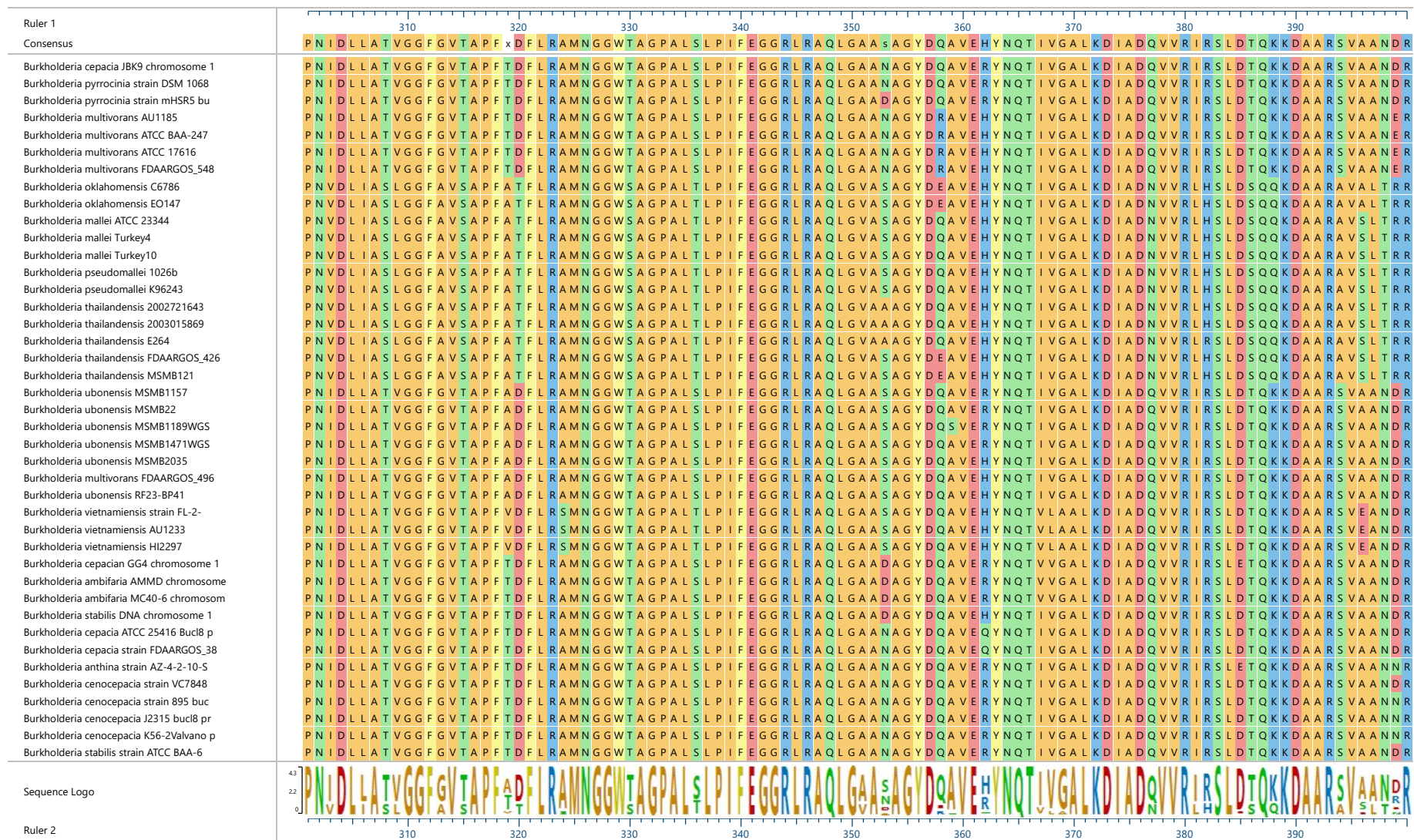

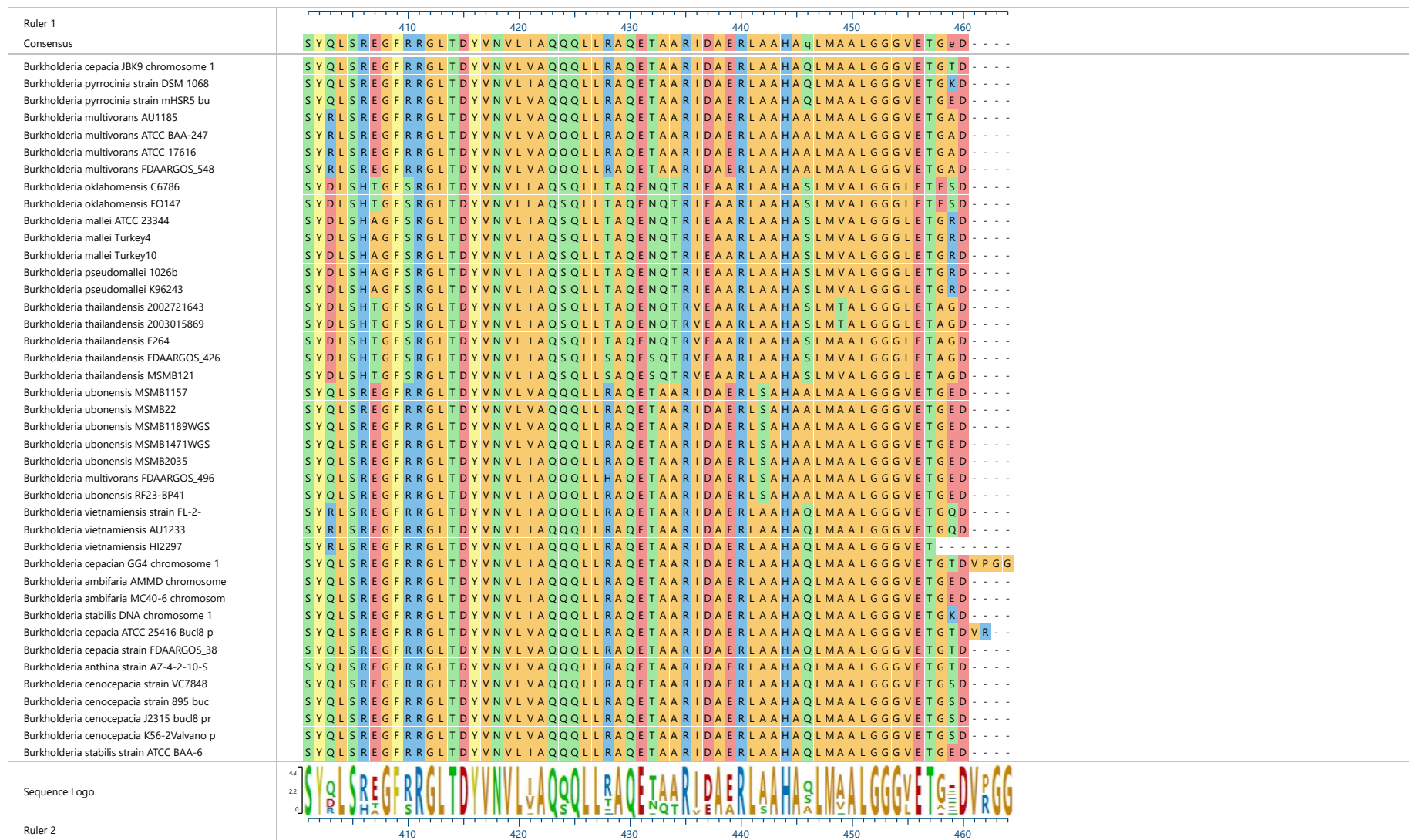

Supplement: Supplementary file 1 [file cells-10-00495-s001.pdf]
